# A Victim of Trances

**Published:** 1889-03

**Authors:** 


### A VICTIM OF TRANCES IN BALTIMORE.

### ASSUMING THE APPEARANCE OF THE DEAD WHOM SHE REPRESENTS.

Psychologists and believers in spiritualism have become interested in Annie Stidham, the sixteen-year-old daughter of Richard Stidham, of Baltimore, who has developed remarkable powers. The family are Roman Catholics. A private exhibition was given the other evening in a brilliantly lighted room, and this afternoon the girl, in the presence of half-a-dozen persons, went into a trance and was said to have communications with the spirits of relatives long since dead. She is a pleasant, hearty looking girl; but this afternoon, when said to be under the influence of the spirit of an old woman who had died of paralysis, the horrible change that came over her countenance was as startling as the transition in the play of "Dr. Jekyll and Mr. Hyde." Her cheeks and temples became pinched and sharp, her lower jaw relaxed. Her chin and nose became pinched and sharp, and with her left hand she slowly stroked the back of her right, with that peculiar motion seen in persons partially paralyzed. Toward the close of the trance she looked like dead. Her father laid her upon the floor. The reporters tried to change the position of her head, feet and hands, but they were immovable as in death. If it was acting, it surpassed the ability of any great actor in feigning death.—*New York Tribune.*

